# Supplementary material for: Honeybee Cognition as a Tool for Scientific Engagement
Source: Insects. 2021 Sep 18;12(9):842. doi: 10.3390/insects12090842 (PMC8471026; doi:10.3390/insects12090842)
Supplement: Supplementary file 1 [file insects-12-00842-s001.zip › insects-1335320- supplementary Table S1.pdf]

insects

MDPI

Article

Honeybee Cognition as a Tool for Scientific Engagement

Jai A. Denton [1,2], Ivan Koludarov [3], Michele Thompson [4], Jarosław Bryk [5] & Mariana Velasque [1,*]

1 Genomics & Regulatory Systems Unit, Okinawa Institute of Science & Technology, Tancha, Okinawa, Japan
2 Institute of Vector-borne Disease, Monash University, Clayton, Victoria, Australia
3 Animal Venomics Group, Justus Liebig University, Giessen, Hessen, Germany
4 Independent Researcher, Dexter, Michigan USA
5 School of Applied Sciences, University of Huddersfield, Huddersfield, UK
* Correspondence: mariana.velasque@oist.jp

Supplementary Data.

Table S1. Number of trainings trails until individual honeybees associated the lemon scent with a sugary reward.

Group ID.	Replicate.	Control.	Dopamine.	Caffeine..
1.	1.	4.	6.	4..
1.	2.	8.	5.	5..
1.	3.	6.	6.	NA..
2.	1.	4.	5.	5..
2.	2.	NA.	5.	5..
2.	3.	NA.	5.	7..
3.	1.	6.	4.	4..
3.	2.	8.	6.	NA..
3.	3.	5.	8.	NA..
4.	1.	NA.	NA.	4..
4.	2.	5.	6.	6..
4.	3.	NA.	8.	5..
5.	1.	6.	NA.	6..
5.	2.	4.	5.	4..
5.	3.	5.	4.	5..
6.	1.	4.	4.	NA..
6.	2.	NA.	4.	5..
6.	3.	NA.	NA.	4..
7.	1.	5.	7.	NA..
7.	2.	6.	6.	4..

7.	3.	4.	NA.	6..
8.	1.	7.	8.	4..
8.	2.	7.	6.	NA..
8.	3.	5.	NA.	4..
9.	1.	5.	5.	5..
9.	2.	4.	6.	7..
9.	3.	5.	4.	5..
10.	1.	6.	7.	NA..
10.	2.	NA.	4.	5..
10.	3.	NA.	7.	7..
11.	1.	5.	6.	4..
11.	2.	6.	8.	6..
11.	3.	4.	6.	NA..
12.	1.	4.	7.	5..
12.	2.	NA.	7.	5..
12.	3.	4.	5.	4..
