# Supplementary material for: Honeybee Cognition as a Tool for Scientific Engagement
Source: Insects. 2021 Sep 18;12(9):842. doi: 10.3390/insects12090842 (PMC8471026; doi:10.3390/insects12090842)
Supplement: Supplementary file 1 [file insects-12-00842-s001.zip › insects-1335320-supplementary File S1.pptx]

## Slide 1
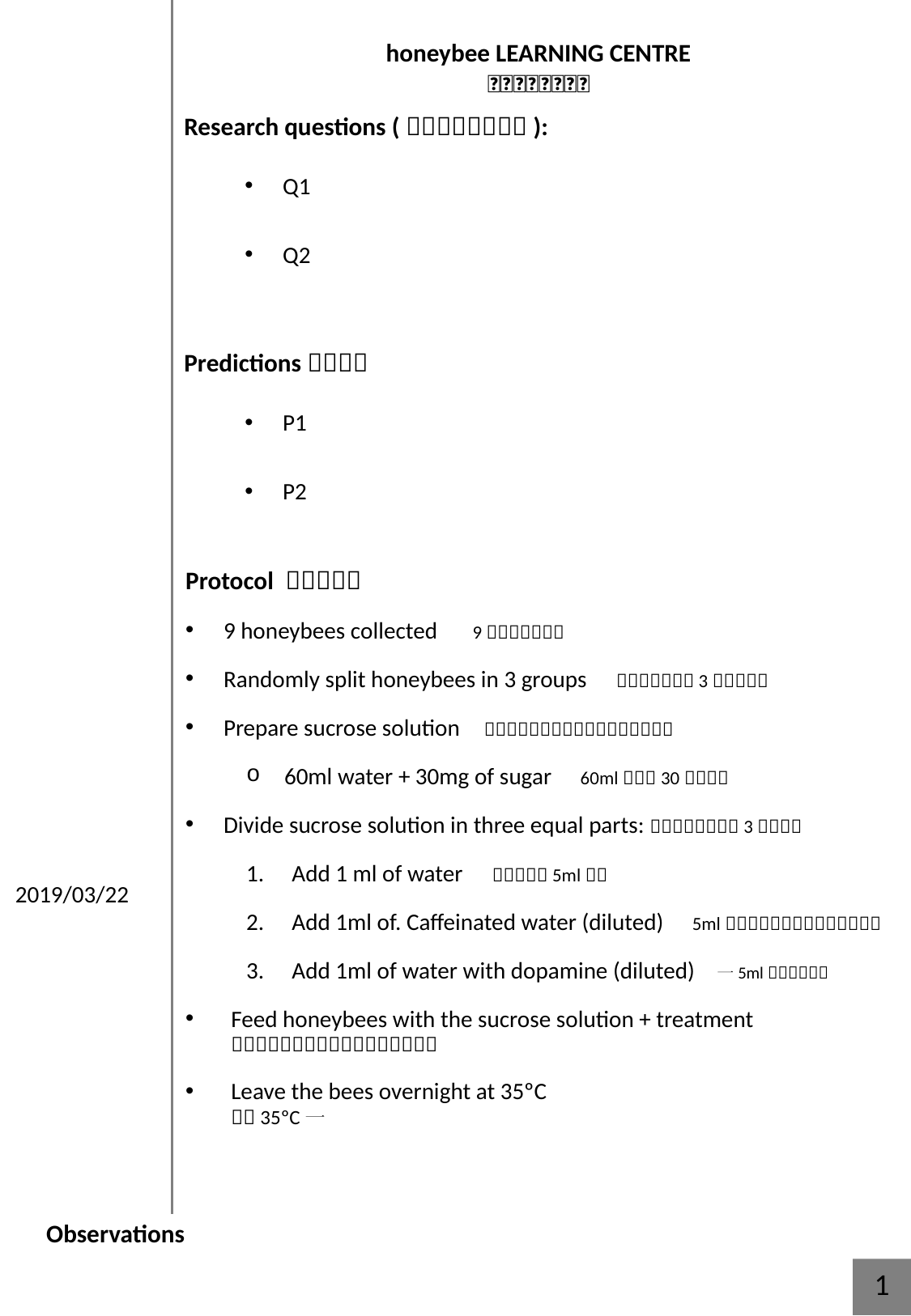

honeybee LEARNING CENTRE
蜜蜂学習センター
Research questions (研究に向けた疑問):
Q1
Q2
Predictions（予測）
P1
P2
Protocol プロトコル
9 honeybees collected　9匹の蜜蜂を回収
Randomly split honeybees in 3 groups　その蜂を適当に3つに分けた
Prepare sucrose solution　スクロース溶液（砂糖水）を準備する
60ml water + 30mg of sugar　60mlの水＋30㎎の砂糖
Divide sucrose solution in three equal parts:スクロース溶液を3等分する
Add 1 ml of water　水をさらに5ml追加
Add 1ml of. Caffeinated water (diluted)　5mlのカフェイン入りの水を加える
Add 1ml of water with dopamine (diluted)　ドーパミンと一緒に5mlの水を加える
Feed honeybees with the sucrose solution + treatment
スクロース溶液を与えられた蜂＋処理
Leave the bees overnight at 35ºC
蜂を35ºCで一晩放置
2019/03/22
Observations
1

## Slide 2
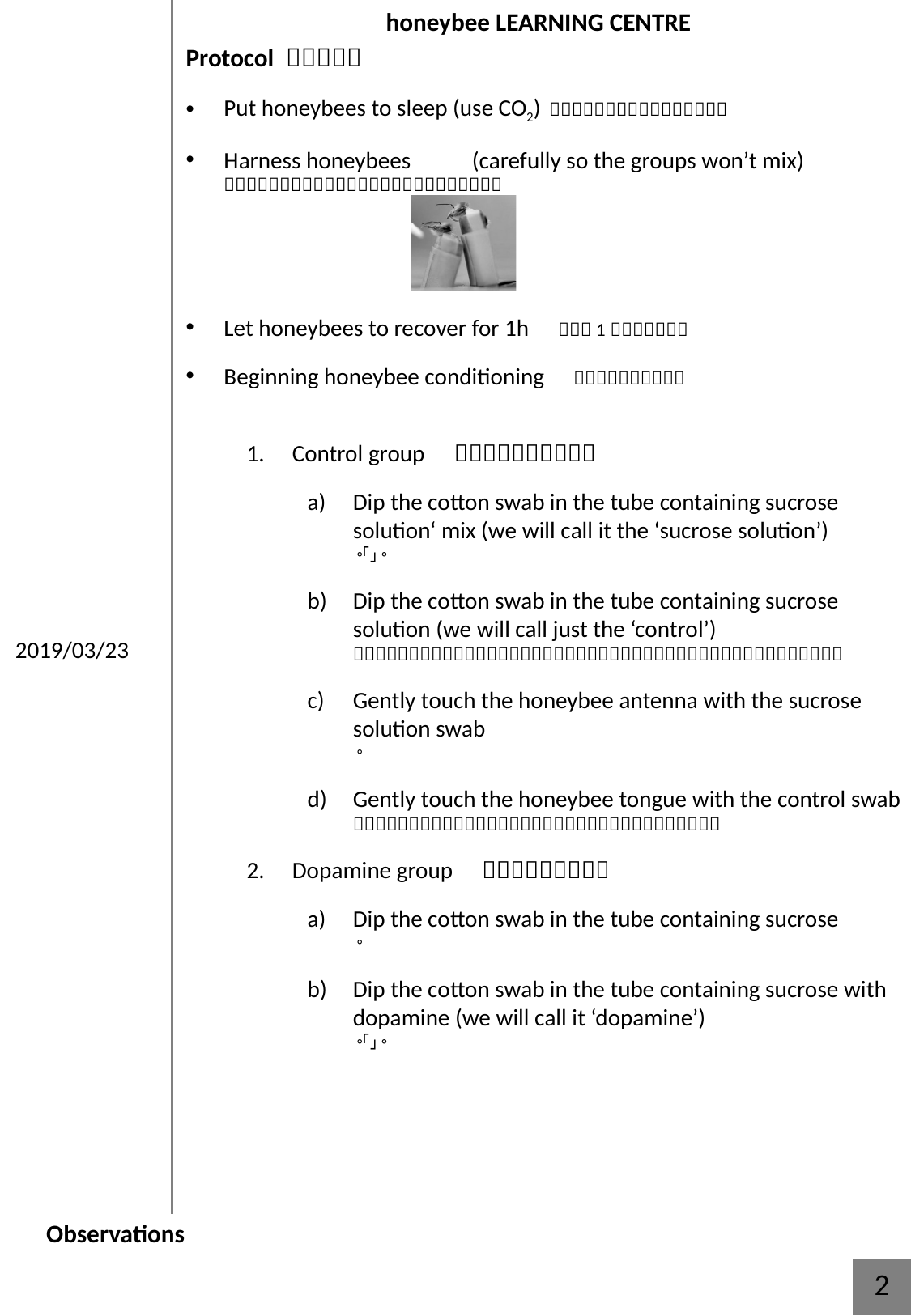

honeybee LEARNING CENTRE
Protocol プロトコル
Put honeybees to sleep (use CO2) 二酸化炭素を使用して蜂を眠らせる
Harness honeybees	 (carefully so the groups won’t mix)
蜜蜂を固定する（慎重にグループが混同しないように）
Let honeybees to recover for 1h　蜜蜂を1時間回復させる
Beginning honeybee conditioning　蜜蜂の条件付けを開始
Control group　コントロールグループ
Dip the cotton swab in the tube containing sucrose solution‘ mix (we will call it the ‘sucrose solution’)
爆発性混合物を含んだチューブに綿棒を浸します。（これを「爆発性」と呼びます）。
Dip the cotton swab in the tube containing sucrose solution (we will call just the ‘control’)
綿棒をスクロース溶液の入ったチューブに浸します（私達は単に「コントロール」と呼びます。）
Gently touch the honeybee antenna with the sucrose solution swab
爆発性の綿棒で蜜蜂のアンテナにそっと触れます。
Gently touch the honeybee tongue with the control swab
コントロール（スクロース溶液）の綿棒でミツバチの舌にそっと触れます
Dopamine group　ドーパミングループ
Dip the cotton swab in the tube containing sucrose
爆発性混合物を含むチューブに綿棒を浸します。
Dip the cotton swab in the tube containing sucrose with dopamine (we will call it ‘dopamine’)
ドーパミンを含んだスクロース溶液に綿棒を浸します。（これを「ドーパミン」と呼びます）。
2019/03/23
Observations
2

## Slide 3
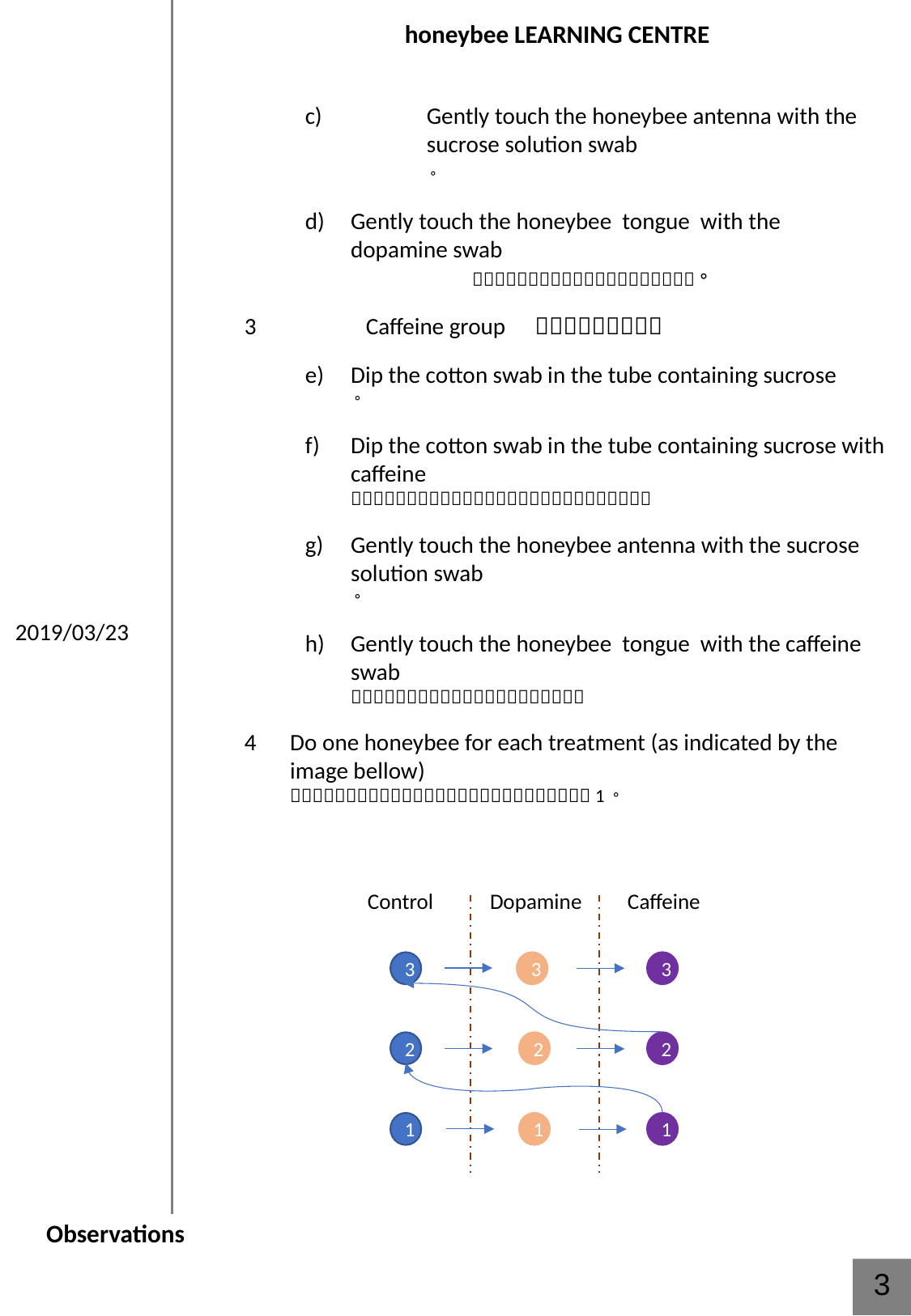

honeybee LEARNING CENTRE
c)	Gently touch the honeybee antenna with the 	sucrose solution swab
	爆発性の綿棒で蜜蜂のアンテナにそっと触れます。
Gently touch the honeybee tongue with the 	dopamine swab
	ドーパミン綿棒で蜜蜂の舌にそっと触れます。
3	Caffeine group　カフェイングループ
Dip the cotton swab in the tube containing sucrose
爆発性混合物を含むチューブに綿棒を浸します。
Dip the cotton swab in the tube containing sucrose with caffeine
カフェインとスクロースを含むチューブに綿棒を浸します。
Gently touch the honeybee antenna with the sucrose solution swab
爆発性の綿棒で蜜蜂のアンテナにそっと触れます。
Gently touch the honeybee tongue with the caffeine swab
カフェイン綿棒で蜜蜂の舌にそっと触れます。
Do one honeybee for each treatment (as indicated by the image bellow)
（下の画像が示すように）それぞれのトリートメントを蜜蜂1匹ずつに対行ってください。
2019/03/23
Control
Dopamine
Caffeine
3
3
3
2
2
2
1
1
1
Observations
3

## Slide 4
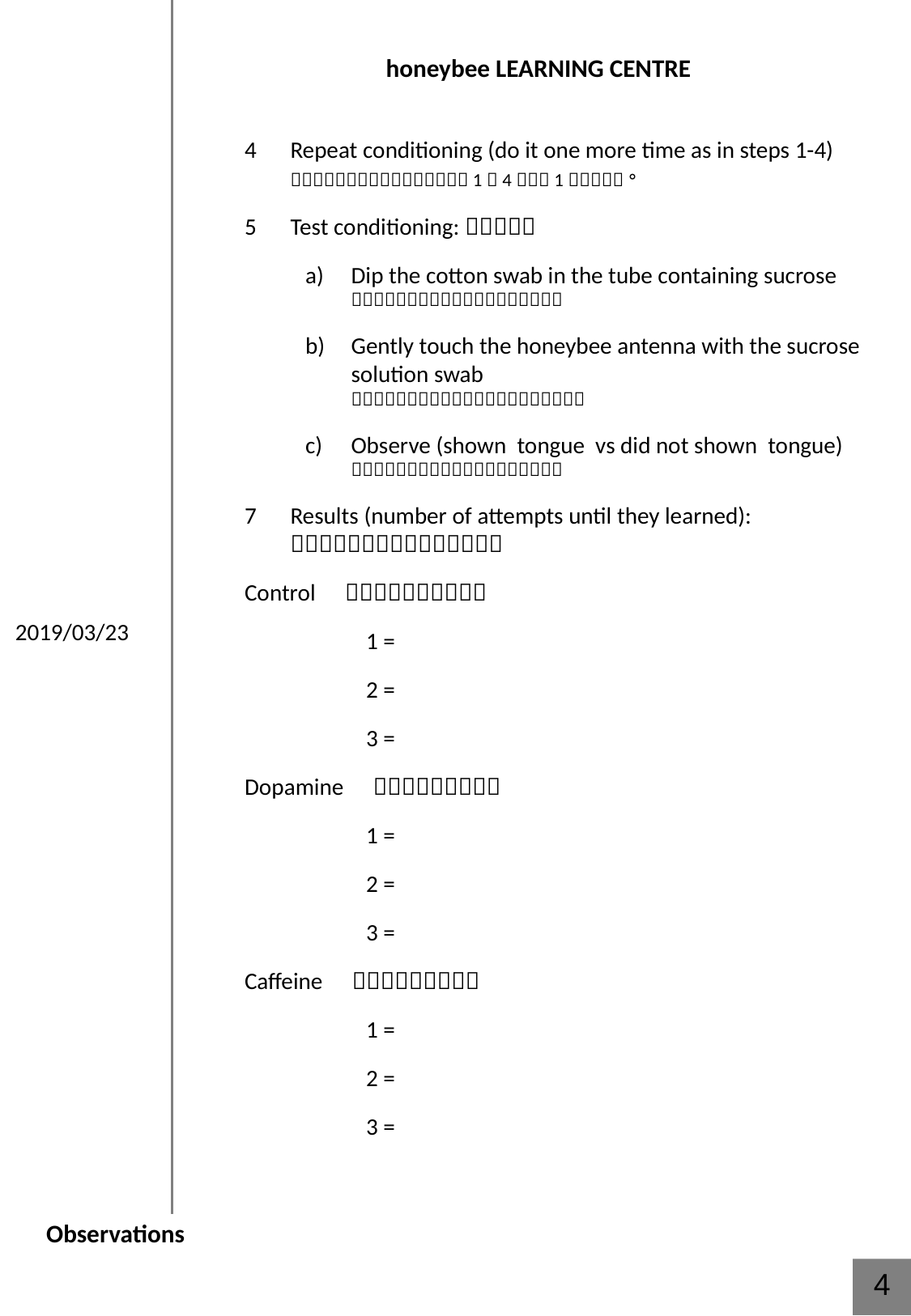

honeybee LEARNING CENTRE
Repeat conditioning (do it one more time as in steps 1-4)
条件付けを繰り返します（ステップ1〜4をもう1回行います）。
Test conditioning:テスト調整
Dip the cotton swab in the tube containing sucrose
爆発性混合物を含むチューブに綿棒を浸す
Gently touch the honeybee antenna with the sucrose solution swab
爆発性の綿棒で蜜蜂のアンテナにそっと触れる
Observe (shown tongue vs did not shown tongue)
観察する（舌を見せる？舌を見せない？）
Results (number of attempts until they learned):
結果（学習するまでの試行回数）
Control　コントロールグループ
	1 =
	2 =
	3 =
Dopamine　ドーパミングループ
	1 =
	2 =
	3 =
Caffeine　カフェイングループ
	1 =
	2 =
	3 =
2019/03/23
Observations
4
